# PPAR Ligands Induce Antiviral Effects Targeting Perturbed Lipid Metabolism during SARS-CoV-2, HCV, and HCMV Infection

**DOI:** 10.3390/biology11010114

**Published:** 2022-01-11

**Authors:** Marialuigia Fantacuzzi, Rosa Amoroso, Alessandra Ammazzalorso

**Affiliations:** Medicinal Chemistry Unit, Department of Pharmacy, G. d’Annunzio University of Chieti-Pescara, Via dei Vestini, 66100 Chieti, Italy; marialuigia.fantacuzzi@unich.it (M.F.); rosa.amoroso@unich.it (R.A.)

**Keywords:** PPAR, viral infection, antiviral drug, lipid metabolism, SARS-CoV-2, HCV, HCMV

## Abstract

**Simple Summary:**

The current coronavirus disease 2019 pandemic turned the attention of researchers to developing novel strategies to counteract virus infections. Despite several antiviral drugs being commercially available, there is an urgent need to identify novel molecules efficacious against viral infections that act through different mechanisms of action. In this context, our attention is focused on novel compounds acting on nuclear receptors, whose activity could be beneficial in viral infections, including coronavirus, hepatitis C virus, and cytomegalovirus.

**Abstract:**

The manipulation of host metabolisms by viral infections has been demonstrated by several studies, with a marked influence on the synthesis and utilization of glucose, nucleotides, fatty acids, and amino acids. The ability of virus to perturb the metabolic status of the infected organism is directly linked to the outcome of the viral infection. A great deal of research in recent years has been focusing on these metabolic aspects, pointing at modifications induced by virus, and suggesting novel strategies to counteract the perturbed host metabolism. In this review, our attention is turned on PPARs, nuclear receptors controlling multiple metabolic actions, and on the effects played by PPAR ligands during viral infections. The role of PPAR agonists and antagonists during SARS-CoV-2, HCV, and HCMV infections will be analyzed.

## 1. Introduction: Targeting Metabolism during Viral Infections

**Figure 1:**
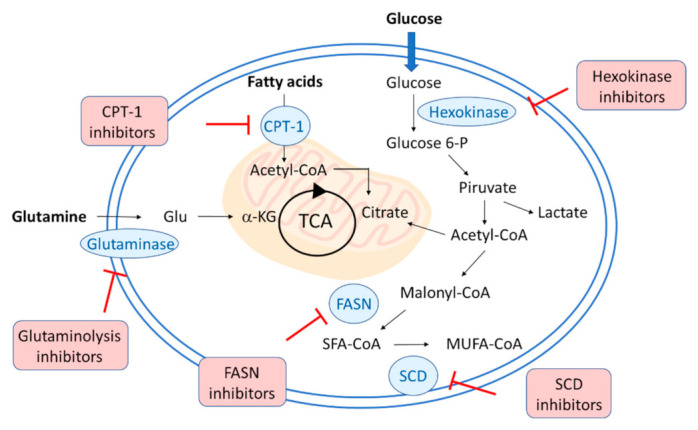
Schematic representation of the metabolic strategies to target viral replication. Hexokinase inhibitors interfere with glycolysis and glucose metabolism, whereas glutaminolysis inhibitors block the glutamate formation from glutamine. The lipid metabolism can be affected with different strategies: carnitine palmitoyl transferase 1 (CPT1) inhibitors block the entry of long-chain fatty acids into mitochondria for oxidation, fatty acid synthase (FASN or FAS) and stearoyl-CoA desaturase (SCD) inhibitors strongly interfere with lipogenesis.

The viral reproduction needs the host cell machinery for the synthesis of viral components, such as nucleic acids and proteins. Clathrin-mediated endocytosis or micropinocytosis are the mechanisms allowing the internalization of viruses in the host, which are subsequently released in the cytosol. At this stage, the viral genome is released and transported into sites where the viral replication occurs [1]. After the replication of viral genome and the subsequent assembly of novel particles (virions), virus leaves from the host cell by exocytosis or lysis [2]. The metabolic capacity of the host cell is of primary importance to ensure the formation of novel virus particles, by supplying the necessary metabolites as amino acids, fatty acids, and nucleotides. Many viruses can manipulate the host metabolism to optimize their biosynthetic needs. The multiple effects played by viruses, involving glucose, lipid, and glutamine metabolism, were extensively reviewed by Eisenreich et al. [3]. The understanding of the nature of metabolic changes imposed by a viral infection is of paramount importance to identify a targeted therapy and obtain a successful outcome. For example, during the human immunodeficiency virus (HIV) infection, the inhibition of glucose metabolism determines the viral elimination, while during the late stages of HIV infection, the intervention on lipid metabolism has been proposed as a fruitful antiretroviral strategy [4,5]. Interestingly, the nutrition status plays a key role in influencing the outcome of viral infections: it is well known that malnutrition, especially a marked reduction in calorie intake, increases the susceptibility of children to viral diseases. Several studies have been conducted in animals to analyze the effects of diet supplementation, mainly vitamins, fat, fibers, and amino acids, during viral infections [6,7,8,9]. Several metabolic strategies have been proposed to target the viral replication in cell cultures and in vivo models. Figure 1 summarizes the main metabolic approaches pursued to manipulate virus metabolism, affecting the glucose, lipid, and glutamine metabolic pathways.

**Figure 2:**
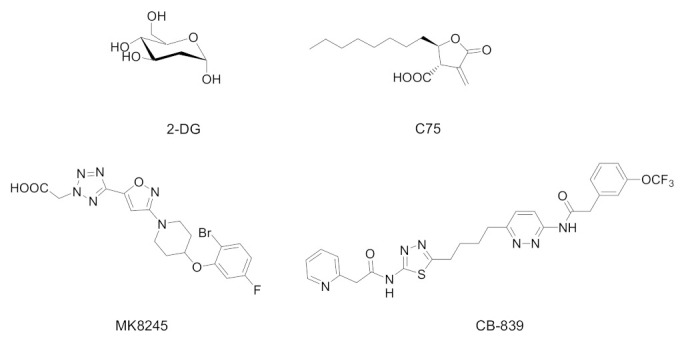
Chemical structures of selected metabolic drugs tested to contrast the virus replication: 2-deoxy-D-glucose (2-DG, phosphoglucose isomerase inhibitor), C75 (FAS inhibitor), MK8245 (SCD1 inhibitor), and CB-839 (glutaminase inhibitor).

Among several metabolic compounds tested for their antiviral activity, promising results were obtained with 2-deoxy-D-glucose (2-DG), which targets glucose metabolism, C75 and MK8245, which interferes with fatty acid metabolism, and CB-839, with its action on glutamine metabolism (Figure 2).

2-DG is a synthetic glucose analog in which the 2-hydroxyl group is replaced by hydrogen. It undergoes an in vivo phosphorylation to 2-deoxy-D-glucose-6-phosphate, which remains trapped into cells, being unable to form its isomer fructose-6-phosphate. The intracellular accumulation of 2-deoxy-D-glucose-6-phosphate leads to the inhibition of glycolysis and glucose metabolism. 2-DG has been attracting interest also as a potential anticancer molecule, thanks to its non-toxic profile and its oral bioavailability [10,11,12]. The influence of 2-DG was studied in different viral infections, such as herpes simplex virus (HSV) [13], influenza virus [14], cytomegalovirus [15], rhinovirus [16]; very recently it has been proposed as an adjuvant therapeutic agent for COVID-19 treatment due to its effects on glycolysis, anti-inflammatory action, and interaction with viral proteins [17].

The possibility to interfere with fatty acid metabolism received great attention, considering it as an attractive target to regulate various and complex diseases as obesity, diabetes, cancer, and cardiovascular complications [18,19]. FAS (or FASN), a lipogenic enzyme catalyzing the condensation of acetyl-CoA and malonyl-CoA to produce long-chain fatty acids, has been proposed as a potential therapeutic target for an antiviral therapy. C75 is a synthetic FAS inhibitor, explored in many studies for its antitumor [20,21] and anti-obesity activities [22,23]. While fatty acid upregulation promotes the synthesis of lipid droplets, the inhibition of the fatty acid biosynthesis provides a mechanism by which the replication of several viruses can be decreased. This has been demonstrated for rotavirus replication, as reported by Gaunt et al. [24].

HIV-1 infection increases the intracellular levels of FAS, making this enzyme a novel host dependency factor with the potential to be exploited as an antiretroviral target [5]. In a similar way, it has been demonstrated that FAS inhibition impairs the replication of respiratory syncytial virus and other respiratory viruses [25].

FAS is upregulated during hepatitis C virus (HCV) infection and regulates the virus entry and production, suggesting that this mechanism could be responsible for the alteration of host cellular lipid profile and causing diseases such as steatosis [26]. The role of FAS in Epstein–Barr virus (EBV) lytic and latent infection has also been explored, showing that FAS plays an important role for the EBV lytic replication [27]. A recent paper describes the effects of C75 in coxsackievirus B3-infected human cells, with a significant decrease in the virus replication [28].

Stearoyl-CoA desaturase (SCD), an endoplasmic reticulum enzyme that catalyzes the biosynthesis of monounsaturated fatty acids (MUFAs) from saturated fatty acids, attracted a lot of attention for its regulation of multiple metabolic processes controlling cellular metabolism, cell cycle progression, survival, and differentiation. Several studies have demonstrated the involvement of SCD in the promotion of cancer cell proliferation, migration, metastasis, and tumor growth, suggesting this enzyme as a therapeutic target for the treatment of cancer [29,30]. While SCD has been shown to be a crucial factor in the lipid metabolism and body weight control, its inhibitors are claimed to be beneficial for the treatment of different diseases, such as skin disorders, nonalcoholic steatohepatitis (NASH), HCV, Alzheimer’s disease, or cancer [31].

There is an emerging body of evidence that the lipid pathways play an important role in HCV’s formation of membranous replication complexes. Since it has been shown that inhibiting lipogenesis negatively affects HCV replication, SCD1 inhibition may also be a novel therapeutic strategy for the treatment of HCV infection [32,33]. The SCD inhibitor MK8245 has been evaluated as an option for anti-HCV multidrug therapy, showing a low risk of emergence of drug-resistant HCV and any significant side effects [34]. Analogously, this compound has been studied against Dengue virus (DENV) infection; it dramatically induced a dose-dependent suppression of DENV replication, without producing cytotoxicity. Interestingly, its efficacy was observed against four DENV serotypes and other flaviviruses, including Zika virus and Japanese encephalitis virus [35].

The glutamine metabolism represents another accessible step for inhibition to obtain a reduction in viral infections. Among the different proposed strategies, targeting glutaminolysis via glutaminase inhibition emerged as a promising strategy to interfere with metabolism. The first step of glutaminolysis, the conversion of glutamine to glutamate, can be reduced by glutaminase inhibitors, which also determine the rise of intracellular ROS levels, and the impairment of GSH production. Due to the critical role of glutaminolysis in cancer metabolism, glutaminase has been proposed as a viable target for cancer therapy [36]. Among several glutaminase inhibitors identified, allosteric inhibitors, such as CB-839 (telaglenastat), appear advantageous, as they selectively target glutaminase without affecting other aspects of glutamine metabolism. CB-839 displayed significant antitumor activity in triple negative breast cancer models [37], head and neck cancer squamous cell carcinoma (HNSCC) [38], hematological malignancies [39,40], and it is currently under clinical trials in solid tumors and in advanced myelodysplastic syndrome. The possibility to use CB-839 in viral infections has been recently explored; CB-839 treatment reduced the viral replication of adenovirus, HSV-1, and influenza A in cultured primary cells [41].

## 2. Changes in Lipid Metabolism during SARS-CoV2, HCV, and HCMV Infection

Lipid metabolism plays a crucial role during the different stages of viral infection, representing an adaptive metabolic response of host organisms. Modifications of lipid metabolism are related to fatty acid metabolism, biosynthesis of sterols and phosphoinositides, and utilization of lipid stores. Many viruses induce high glucose metabolism (Warburg effect) and change the lipid metabolism from fatty acid oxidation (FAO) to fatty acid synthesis. The increase in FAS is mainly necessary for enveloped viruses such as COVID-19, influenza, hepatitis B and C and others [42,43]. Metabolic changes induced by virus infection provide potential target to contrast the infection. The outcome of viral infections is strongly related to the metabolic conditions of host, including obesity, diabetes mellitus, metabolic syndrome, and endocrine diseases. In addition, the state of nutrition has been shown an important factor in determining the evolution of viral infections, by altering multiple metabolic pathways.

HCV infection, the main cause of hepatitis, cirrhosis, and hepatocellular carcinoma, exerts a strong influence on host lipid metabolism [44]. Consequently, hypolipidemia and hepatic steatosis are conditions frequently occurring in chronic hepatitis C patients. These metabolic changes in host highlight the close interplay between HCV and the lipid metabolism, with lipids and lipoproteins playing a crucial role in the virus lifecycle [45]. A strict relationship between lipids and HCV is necessary for the mechanism of viral entry into hepatocytes, replication, particles assembly and secretion [46]. While lipids and their receptors are key players in the early stages of HCV infection, molecules targeting lipids or their receptors could be considered as novel strategies against HCV infection, by preventing or limiting virus-induced damages [47]. Likewise, HCV, hepatitis B virus (HBV), is considered a metabolic virus, as it affects many hepatic metabolic pathways [48]. Several studies based on cell lines and mouse models explored the mechanisms by which HBV infection affects lipid metabolism but, to date, results are not conclusive. HBV virus induces extensive changes in hepatic lipid metabolism, by activating the expression of some proteins or upregulating fatty acid oxidation. For example, the HBV infection induces sterol regulatory element-binding protein 1c (SREBP1c) activation, with upregulation of lipogenic enzymes, such as FAS, SCD, acetyl-CoA carboxylase (ACC), and thus fatty acid synthesis [49].

Several studies on members of the Coronaviridae family demonstrated the importance of lipids in different steps of the virus lifecycle, although the exact mechanisms need to be fully elucidated [50]. The early stage of coronavirus infection includes events prior to the viral RNA replication and involves the virus fusion with host membrane and endocytosis, whereas the late stage involves the RNA replication, the synthesis of proteins, the viral particle assembly and release. Following the early interaction with membrane lipids, such as cholesterol, the internalized virus leads to an extensive reorganization of host lipid profile, necessary to the genome replication of virus. It is not surprising that a specific lipid profile, perturbed by the virus action, is requested to ensure a productive infection.

Human cytomegalovirus (HCMV) is a common herpesvirus that causes lifelong persistent infections. While infection is asymptomatic in most people, HCMV induces life-threatening illnesses in immunocompromised people, mainly in transplant recipients and cancer patients. In addition, HCMV infection is a leading cause of congenital disabilities. HCMV replication is strongly related to host metabolism; in fact, the CMV infection alters the activity in multiple metabolic pathways, including fatty acid elongation and lipid synthesis. The regulation of multiple factors in the host organism, such as kinases and transcription factors, are important for metabolic changes occurring after HCMV infection. A comparative study was carried out to assess the influence of two enveloped viruses, HCMV and HSV-1, on host metabolism [51]. It is interesting to observe that both viruses do not rely passively on host metabolic activity, but they actively redirect metabolism, by following different metabolic programs. HCMV determines an increased lipid metabolism, raising the glycolytic flux, FAS, lactate excretion, and increasing the levels of many molecules involved in TCA cycle. Differently from HCMV, HSV-1 shows less reliance on FAS, while its envelope is mainly formed from preexisting cellular material, whereas it upregulates flux from glucose to de novo pyrimidine nucleotide biosynthesis. These divergent metabolic strategies represent an important point to interfere with metabolic changes induced by viruses, allowing us to point out different metabolic processes to obtain novel antiviral strategies.

A recent study indagated on viral factors required to alter metabolism, by identifying the HCMV UL37x1 protein (pUL37x1) as a key factor for infection-associated increases in lipid metabolism [52]. This protein was found to be crucial for the increased lipid metabolism, being involved in fatty acid elongation to synthesize very long chain fatty acids (VLCFAs). The HCMV infection resulted also in a sensible increase in phospholipids, mainly those possessing VLCFA tails. Through a combined metabolomic and lipidomic approach, this study demonstrated the impact of the viral UL37x1 protein in remodeling host lipid metabolism.

## 3. PPARs in Viral Infections

Peroxisome Proliferator-Activated Receptors (PPARs), belonging to the nuclear receptor superfamily, are transcription factors playing well-established roles in several metabolic pathways in the organism, including lipid and glucose metabolism, energetic homeostasis, cell differentiation and proliferation. Since their discovery, a body of knowledge has been collected on them, and the three receptor subtypes (PPARα, PPARγ, PPARβ/δ) have attracted a great deal of efforts by medicinal chemists to identify novel drugs targeting the metabolism. PPAR agonists, antagonists, and modulators represent important pharmacological tools to induce beneficial therapeutic effects in metabolic disorders, such as metabolic syndrome, obesity, diabetes, and others [53,54,55,56]. Recently, PPARs have been attracting a great deal of interest as targets in different cancers, thanks to their ability to induce important metabolic changes in tumors, and several PPAR ligands have been studied for their possible antitumor effects [57,58,59,60].

The involvement of PPARs in viral infections is quite a recent matter of investigation, and the interest in this field received a strong impulse due to the pandemic by COVID-19. PPAR agonists, antagonists, or modulators could induce different effects in models of infections; in the following sections, the main results obtained from in vitro and in vivo studies will be analyzed and discussed. In addition, PPAR modulators may act as agonist or antagonist depending on the cellular context, based on the variable presence of coactivators or corepressors in specific tissues. Unfortunately, it is currently impossible to precisely categorize the effects of PPAR ligands in viral infections, so as identify the exact mechanism of action responsible for the observed antiviral outcome. In next paragraphs main evidence on PPAR involvement in the most studied infections (SARS-CoV-2, HCV, and HCMV) will be analyzed, focusing on the description of PPAR ligands and the beneficial effects observed in models of infection.

### 3.1. Antiviral Effects Played by PPAR Ligands in SARS-CoV-2 Infection

The COVID-19 pandemic is an ongoing global problem caused by severe acute respiratory syndrome coronavirus 2 (SARS-CoV-2). A very large body of studies has been conducted on this subject, with a recent investigation demonstrating that cell lipid profile is significantly altered during the infection by human coronavirus [61]. COVID-19 progression has been suggested to have a metabolic origin, while elevated glucose and lipid levels are risk factors. The SARS-CoV-2 spike protein mediates the viral cellular entry via the ACE2 receptor. Recent findings have revealed that ACE2 is downregulated in SARS-CoV-2-infected lung tissue [62]. The stimulation of ACE2 could play a protective role in the treatment of hypertension, heart disease, cancer, and COVID-19 [63], while these disorders show the upregulation of the WNT/β-catenin pathway. For these reasons, pharmacological agents increasing the ACE2 expression, such as statins and PPARγ agonists, could be beneficial in the treatment of COVID-19.

**Figure 3:**
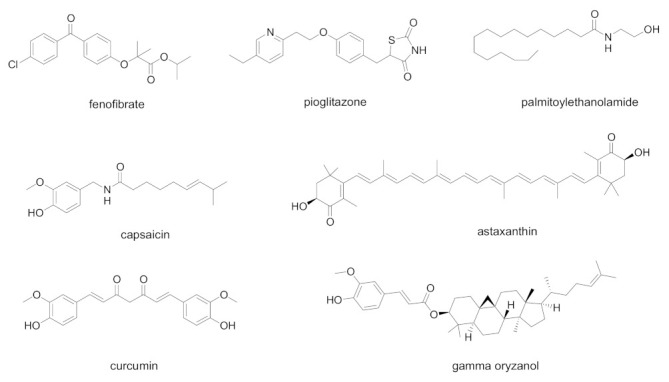
Chemical structures of natural and synthetic PPAR ligands explored in in vitro assays or proposed as possible antiviral agents against SARS-CoV-2 infection.

In Figure 3 are summarized the chemical structures of natural and synthetic PPAR ligands explored in in vitro assays or proposed as possible antiviral agents against SARS-CoV-2 infection.

Emerging studies suggest that SARS-CoV-2 alters lipid metabolism in the lung epithelial cells by modulating PPARα, possibly contributing to lipotoxicity and respiratory effects. The reduced PPARα activity could be responsible for the pulmonary inflammation, playing an important role in the pathogenesis of acute lung injury. For these reasons, the use of PPARα agonists may be a useful therapeutic strategy to reverse the inflammatory and metabolic changes induced by SARS-CoV-2 [64]. A recent study showed that the PPARα agonist fenofibrate reversed the metabolic changes caused by SARS-CoV-2 and inhibited the viral replication in lung epithelial cells [65]. Fenofibrate was found to inhibit the cytopathic effect exerted by SARS-CoV-2 on Vero E6 cells at 20 µM [66]. The PPARα agonist palmitoylethanolamide (PEA) has well-known anti-inflammatory effects, and a very recent study identified the putative mechanisms by which this compound could represent a promising adjuvant in the current COVID-19 therapeutic protocols [67]. By the selective activation of PPARα, in primary cultures of murine alveolar macrophages PEA antagonizes the nuclear factor-κB (NF-κB) signalling pathway, decreasing the production of tumor necrosis factor alpha (TNF-α), interleukin β (IL-1β) and other inflammatory mediators.

PPARγ has been appointed as a promising target for the treatment of viral influenza associated with the inflammatory pathways occurring during the cytokine storm. By targeting inflammation, ACE2 and the WNT/β-catenin pathway, PPARγ agonists may be interesting candidates for delivering SARS-CoV-2 therapy in clinical settings [68]. The stimulation of PPARγ by natural or synthetic agonists could exert a regulatory role on the cytokine storm typical of virus infections, by preventing the cytokine overproduction and the inflammatory cascade. Natural PPARγ ligands, including curcumin, capsaicin, docosahexaenoic acid, eicosapentaenoic acid, have been proposed for possible use in COVID-19 [69].

The use of PPARγ agonist pioglitazone was recently analyzed in people affected by type 2 diabetes for the risk of increased morbidity and mortality in COVID-19 infection [70]. By considering the literature data, pioglitazone has more potential for benefit than harm, and can be continued in diabetic people with mild/moderate COVID-19. In addition, pioglitazone has been indicated as a potential 3-chymotrypsin-like protease (3CL-Pro) inhibitor, which could downregulate SARS-CoV-2 RNA synthesis and replication [71]. This interesting concept needs further investigation to assess the real role of pioglitazone during SARS-CoV-2 infection. Pioglitazone has been also proposed as an effective treatment in COVID-19 patients with diabetes, hypertension, and cardiovascular comorbidities, thanks to its ability to reduce inflammatory parameters [72].

Natural compounds have also been investigated as possible coadjuvant therapy to prevent the cytokine storm in COVID-19 patients with obesity conditions. Gamma-oryzanol, the main bioactive constituent from rice bran and germ, has been found to positively modulate PPARγ expression in adipose tissue, reducing the levels of TNF-α, interleukin-6 (IL-6) and monocyte chemoattractant protein-1 (MCP-1) [73].

For its potent and multiple PPAR activity, astaxanthin was studied as an effective therapeutic strategy to regulate the host inflammatory and immune responses, contrast the cytokine storm, and prevent the inflammatory effects following COVID-19 [74]. Several in vitro and in vivo studies related to astaxanthin showed beneficial effects in alleviating the risk of inflammatory cytokine, thereby supporting its likely therapeutic benefits against cytokine storm in COVID-19 infection. However, to date, the antiviral action of astaxanthin requires further investigation.

Interestingly, a recent study proposes PPARα and PPARγ agonists as adjuvants for COVID-19 vaccine, thanks to their ability to enhance both B and T memory cells through different mechanisms [75].

To the best of our knowledge no studies have been reported to date concerning the involvement of PPARδ on COVID-19 infection and the possible beneficial effects induced by PPARδ selective agonists or antagonists. Overall, results from literature studies suggest the lipid metabolism regulation through PPARα and PPARγ would be a druggable target against coronavirus infections. In Table 1 results from published studies are summarized, pointing at PPAR ligands, concentration, infected cell models, and observed outcome in SARS-CoV-2 infection. In addition, PPAR ligands proposed as anti-SARS-CoV-2 agents are reported, based on their anti-inflammatory, antioxidant, and immunomodulating properties.

Despite the evidence arising from the studies reviewed, a clear understanding on the real suitability of the clinic use of PPAR drugs during COVID-19 infection is so far yet to be elucidated. PPAR ligands, such as glitazones, can also induce biological effects through a PPARγ-independent mechanism, as widely demonstrated for cytotoxic effects in different in vitro cancer models [76]. The cytotoxicity induced by troglitazone in human renal cell carcinoma cell lines has been demonstrated to be independent by PPARγ pathway [77]; analogously, the antiproliferative effect induced by fenofibrate in human hepatoma cell line Huh7 was not related to PPARα stimulation, while this effect was not suppressed by using the selective PPARα antagonist GW6471 [78]. These findings support the high versatility of PPAR drugs in inducing pleiotropic effects, and this aspect is also complicated by the need to reach elevated concentrations of drug to observe the desired pharmacological effect [79].

### 3.2. Antiviral Effects Played by PPAR Ligands in HCV Infection

The role of PPARs in controlling glucose and lipid metabolism in hepatocytes is well established, leading to liver inflammation and fat accumulation, which represent hallmarks of chronic HCV infection. To date, many studies have been carried out to clarify the exact correlation between PPARs and HCV infection [80]. PPARs are highly expressed in hepatocytes in normal conditions, whereas it has been demonstrated an altered liver PPARα and γ expression during HCV infection [81,82,83]. The correlation between PPARα expression and HCV attracted a great deal of attention, this isoform being a major player in the liver lipid homeostasis.

**Figure 4:**
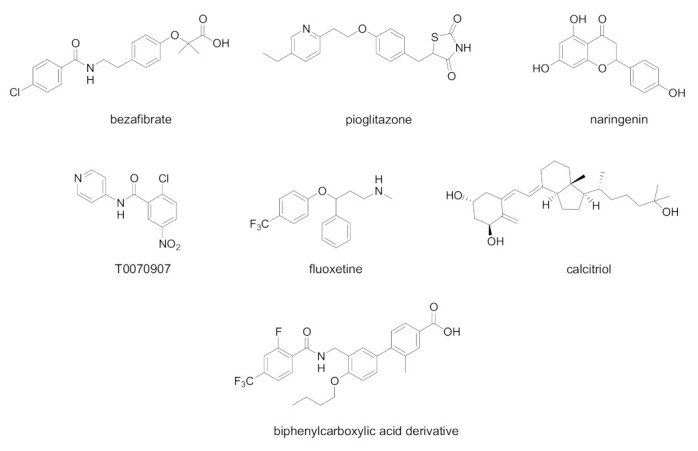
Chemical structures of natural and synthetic PPAR ligands displaying in vivo or in vitro efficacy in HCV infection models.

In Figure 4, chemical structures of natural and synthetic PPAR ligands tested in in vitro or in vivo studies against HCV infection are displayed.

The effects of bezafibrate, a classical PPAR pan agonist, were indagated in pilot studies of chronic HCV infection [84,85,86]. At the endpoint, the viral load and liver enzymes were significantly decreased, suggesting antiviral and anti-inflammatory properties of bezafibrate. An oral treatment with bezafibrate (400 mg/day for 8 weeks) was effective in patients with chronic hepatitis C, by reducing serum HCV RNA. The evidence of an association between HCV and LDL levels in serum suggests the potential usefulness of bezafibrate as an anti-HCV agent.

The PPARγ agonist pioglitazone was found to induce a similar effect, by producing a decrease in the HCV viral load [87]. A pilot study was conducted on overweight genotype 4 HCV-infected patients from Egypt; the treatment with pioglitazone at 30 mg/day for 14 days of treatment was effective in decreasing serum HCV RNA at day-14. Pioglitazone treatment decreased also fasting serum glucose, and induced modifications on liver injury indicators, as serum alanine aminotransferase and, to a smaller degree, aspartate aminotransferase levels.

The grapefruit flavonoid naringenin dose-dependently inhibited HCV production blocking the assembly of viral particles [88]. This antiviral effect was in part ascribed to PPARα activation, leading to a decreased VLDL production without causing hepatic lipid accumulation in Huh7.5.1 cells and primary human hepatocytes. Additionally, long-term treatment with naringenin (200 µM for 4 days of treatment) led to a rapid inhibition of HCV production.

An interesting study showed that the antidepressant drug fluoxetine exerts antiviral effects in an HCV model [89]. Fluoxetine could represent a novel treatment for HCV infection through reduction in ROS generation, reduced lipid accumulation and activation of c-Jun amino-terminal kinases (JNK) and PPARγ/δ pathways. PPARγ and PPARδ antagonists reverted the inhibitory effect on HCV infection and lipid accumulation, confirming the involvement of these nuclear receptors in the mechanism of antiviral activity; conversely, PPARα antagonist GW6471 showed no effect.

Interestingly, a recent study shows that the antiviral effects played by calcitriol are associated with a reduced PPAR activation [90]. HCV-infected Huh7.5 human hepatoma cells (MOI 0.01) were treated with various concentrations of calcitriol for 6 days of treatment. By blocking three PPAR isotype activation, calcitriol produced the partial inhibition of HCV infection via PPARα/γ, the relief of nitrative stress via PPARβ/γ and the reduction in lipid accumulation via PPARγ. These effects were reverted in the presence of PPAR agonists Wy14643, Ly171883, and linoleic acid.

The PPARα/γ antagonist 2-chloro-5-nitro-*N*-(pyridyl)benzamide (T0070907) displayed a significant anti HCV effect in Huh-7 cells, related to the ability of this molecule to suppress the PPARα activity [91]. The decrease in viral replication in in vitro studies (IC_50_ 19.1 µM) was linked to the misregulation of lipid homeostasis, leading to increased lipid content. However, the exact mechanism by which PPARα inhibits HCV lifecycle needs to be fully clarified.

The possible antiviral effects played by PPARδ antagonists were also investigated [92]. A group of biphenyl carboxylic acids with PPARδ antagonist properties were found to dose-dependently inhibit HCV RNA replication, indicating that PPARδ pathway is also involved in antiviral effects. The best compound of this series displayed a submicromolar potency (EC_50_ 0.22 µM) and a weak cytotoxicity against host cells. Interestingly, the combinations of this compound with pegylated interferon alpha (Peg-IFNα) or Peg-IFNα and ribavirin were also able to improve the outcome of HCV infection, leading to a synergistic decreased HCV RNA replication.

A summary of PPAR ligands tested in in vivo or in vitro HCV infection models is depicted in Table 2.

### 3.3. Antiviral Effects Played by PPAR Ligands in HCMV Infection

Although infection by HCMV is usually benign, congenital infection is a leading cause of permanent abnormalities of the CNS. In addition, HCMV infection contributes to the development of pathological states in immune-compromised hosts such as AIDS patients and transplant recipients. The relationships between HCMV infection and PPARs have been explored, given the known role of PPARs, mainly PPARγ isotype, in lipogenesis and inflammation. Experiments provided evidence that PPARγ transcriptional activity is induced in HCMV-infected cells, as evidenced by nuclear translocation and lipid accumulation [93]. The PPARγ activation by HCMV is involved in defective trophoblastic migration and invasion processes, being PPARγ a major player in cytotrophoblast differentiation and function.

The effects of HCMV infection in human immortalized extravillous cytotrophoblasts (HIPEC) were also analyzed, identifying 15-hydroxyeicosatetraenoic acid (15-HETE) and 13-hydroxyoctadecadienoic acid (13-HODE) as predominant PPARγ agonists secreted after HCMV infection [94]. The PPARγ stimulation by these natural ligands results in impaired migration abilities and enhanced viral replication, confirming the important role of PPARγ in HCMV infection.

In addition, the role of PPARγ in neurogenesis during congenital HCMV infection was deeply studied in neural stem cells from human embryonic stem cells (NSCs) [95]. This study confirmed a key role for PPARγ in neurogenesis and in the pathophysiology of HCMV infection, displaying that viral infection triggers PPARγ levels in NSCs. Infected cells produce increased levels of 9-hydroxyoctadecadienoic acid (9-HODE), which is responsible for enhanced viral replication and impaired neurogenesis. The treatment of infected NSCs with the PPARγ antagonist T0070907 restored a normal rate of differentiation.

To date, only the relationship of HCMV-PPARγ has been reported in the literature, and details about the use of PPARγ agonists or antagonists are not available. To the best of our knowledge, no studies have been published focusing on the possible effects played by PPARα and PPARδ pathways during HCMV infection.

## 4. Conclusions

The strict interplay existing between virus and host metabolism offers the possibility to interfere with metabolic pathways as an attractive novel strategy to obtain a successful outcome in viral infections. The possibility to target PPARs has received attention in recent years, given the well-established roles of these nuclear receptors in glucose and lipid metabolism. However, to date, a limited number of experimental studies describing PPAR ligands tested in viral infections are available, and they mainly involve SARS-CoV-2, HCV, and HCMV infections. These studies were reviewed in this work, pointing at chemical structures, mechanisms of action on three PPAR isoforms (agonist, antagonist, modulator), and the outcomes observed in in vitro and in vivo models of infection. Unfortunately, it is currently impossible to precisely categorize the effects of PPAR ligands in viral infections, so as to identify the exact mechanism of action responsible for the observed antiviral effects. In SARS-CoV-2 infection, the use of PPAR ligands could induce beneficial effects by preventing the cytokine overproduction and the subsequent inflammatory cascade. Additionally, in HCV, the use of PPAR agonists and antagonists was found to be effective to decrease viral levels in patients or in in vitro cell models. Very little is known about the possibility to use a PPAR ligand to contrast the HCMV infection; however, some studies provide evidence about the relationships between PPARs and HCMV infection, suggesting PPAR ligands as therapeutic options in the management of HCMV-infected patients. A deeper understanding of viral interactions with host metabolism is necessary to provide novel therapeutic options in the fight against viral diseases. A further aspect to be clearly elucidated is to know the exact mechanism of action of PPAR drugs, excluding possible off targets effects.

## Figures and Tables

**Table 1:** Summary of PPAR ligands in vitro tested in SARS-CoV-2 infection or proposed as anti-SARS-CoV-2 agents.

Cpd	PPAR Activity	Concentration	Cell	Outcome	Ref.
Fenofibrate (Tricor^®^)	PPARα agonist	20 µM	Human bronchial epithelial cells	Block viral replication, reverted effects on phospholipid accumulation and glycolysis	[65]
Fenofibrate	PPARα agonist	20 µM	Vero E6 cells	Block viral entry	[66]
Palmitoylethanolamide	PPARα agonist	10^−9^–10^−7^ M	murine alveolar macrophages	Inhibition of NF-κB transcription and NLRP-3 inflammasome signaling, with a significant antinflammatory effect	[67]
Pioglitazone	PPARγ agonist	-	-	Proposed as 3CL-Pro inhibitor, it could downregulate SARS-CoV-2 RNA synthesis and replication Proposed as an additive in COVID-19 patients with diabetes, hypertension, and cardiovascular comorbidities for its antinflammatory properties	[71,72]
Curcumin, capsaicin, docosahexanoic acid, eicosapentaenoic acid	Natural PPARγ agonists	-	-	Proposed for use in COVID-19, due to their ability to prevent cytokine overproduction and inflammatory cascade	[69]
Gamma-oryzanol	PPARγ modulator	-	-	Proposed for use in COVID-19, due to its anti- inflammatory and antioxidant properties	[73]
Astaxanthin	Multiple action on PPARs: PPARα agonist, PPARδ antagonist, PPARγ agonist or antagonist	-	-	Proposed for use in COVID-19, due to the ability to reduce the oxidative stress induced by ROS, the immune response, and the production of pro-inflammatory cytokines	[74]

**Table 2:** Summary of PPAR ligands in vivo or in vitro tested in HCV infection.

Cpd	PPAR Activity	Concentration	Model	Outcome	Ref.
Bezafibrate	PPAR pan agonist	400 mg/day for 8 weeks	Chronic hepatitis C patients complicated with hyperlipidemia	Decreased serum HCV RNA	[86]
Pioglitazone	PPARγ agonist	30 mg/day for 14 days	Overweight Genotype 4 HCV patients	Decreased serum HCV RNA at day 14	[87]
Naringenin	PPARα agonist	200 µM	HCV-infected Huh7.5.1	Inhibition of ApoB-100 and HCV RNA secretion	[88]
Fluoxetine	PPARγ/δ modulator	0.1–10 µM for 6 days	HCV-infected Huh7.5 cells	Decrease in virus protein levels of core, NS3, and NS5A. Reduction in ROS generation and lipid accumulation	[89]
Calcitriol	PPARα/γ/δ modulator	0.1–1000 nM	HCV-infected Huh7.5 cells	Decrease in viral infection, nitrative stress, and lipid accumulation	[90]
T0070907	PPARα/γ antagonist	IC_50_ 19.1 µM	Huh-7 cells expressing an HCV subgenomic replicon	Inhibition of HCV replication	[91]
Biphenylcarboxylic acids	PPARδ antagonists	2.5–10.0 µM, most potent compound EC_50_ 0.22 µM	OR6 HCV replication system	Dose-dependent inhibition of HCV RNA replication. Synergistic antiviral effect when tested in combination with Peg-IFNα or Peg-IFNα and ribavirin	[92]

## Data Availability

Not applicable.

## Abbreviations

13-HODE	13-Hydroxyoctadecadienoic acid
15-HETE	15-Hydroxyeicosatetraenoic acid
2-DG	2-Deoxy-D-glucose
3CL-Pro	3-Chymotrypsin-like protease
9-HODE	9-Hydroxyoctadecadienoic acid
ACC	Acetyl-CoA carboxylase
CPT1	Carnitine palmitoyl transferase 1
DENV	Dengue virus
EBV	Epstein–Barr virus
FAO	Fatty acid oxidation
FAS (or FASN)	Fatty acid synthase
GSH	Glutathione
HBV	Hepatitis B virus
HCMV	Human cytomegalovirus
HCV	Hepatitis C virus
HIPEC	Human immortalized extravillous cytotrophoblasts
HIV	Human immunodeficiency virus
HNSCC	Head and neck cancer squamous cell carcinoma
HSV	Herpes simplex virus
IL-1β	Interleukin 1 beta
IL-6	Interleukin 6
JNK	c-Jun amino-terminal kinases
LDL	Low-density lipoprotein
MCP-1	Monocyte chemoattractant protein-1
MOI	Multiplicity of infection
MUFAs	Monounsaturated fatty acids
NASH	Nonalcoholic steatohepatitis
NF-κB	Nuclear factor-κB
PEA	Palmitoylethanolamide
Peg-IFNα	Pegylated interferon alpha
PPARs	Peroxisome Proliferator-Activated Receptors
pUL37x1	UL37x1 protein
ROS	Reactive oxygen species
SARS-CoV-2	Severe acute respiratory syndrome coronavirus 2
SCD	Stearoyl-CoA desaturase
SREBP1c	Sterol regulatory element-binding protein 1c
TCA	Tricarboxylic acid
TNF-α	Tumor necrosis factor alpha
VLCFAs	Very long chain fatty acids
VLDL	Very-low-density lipoprotein
NSCs	Neural stem cells
